# Deep Learning Methods for Tracking the Locomotion of Individual Chickens

**DOI:** 10.3390/ani14060911

**Published:** 2024-03-15

**Authors:** Xiao Yang, Ramesh Bahadur Bist, Bidur Paneru, Lilong Chai

**Affiliations:** Department of Poultry Science, College of Agricultural & Environmental Sciences, University of Georgia, Athens, GA 30602, USA

**Keywords:** poultry locomotion, deep learning, track anything model, animal welfare, non-intrusive tracking

## Abstract

**Simple Summary:**

Poultry locomotion is an important indicator of animal health, welfare, and productivity. This research introduced an innovative approach that employs an enhanced track anything model (TAM) to track chickens in various experimental settings for locomotion analysis. The model demonstrated notable accuracy in speed detection, as evidenced by a root mean square error (RMSE) value of 0.02 m/s, offering a technologically advanced, consistent, and non-intrusive method for tracking and estimating the locomotion speed of chickens.

**Abstract:**

Poultry locomotion is an important indicator of animal health, welfare, and productivity. Traditional methodologies such as manual observation or the use of wearable devices encounter significant challenges, including potential stress induction and behavioral alteration in animals. This research introduced an innovative approach that employs an enhanced track anything model (TAM) to track chickens in various experimental settings for locomotion analysis. Utilizing a dataset comprising both dyed and undyed broilers and layers, the TAM model was adapted and rigorously evaluated for its capability in non-intrusively tracking and analyzing poultry movement by intersection over union (mIoU) and the root mean square error (RMSE). The findings underscore TAM’s superior segmentation and tracking capabilities, particularly its exemplary performance against other state-of-the-art models, such as YOLO (you only look once) models of YOLOv5 and YOLOv8, and its high mIoU values (93.12%) across diverse chicken categories. Moreover, the model demonstrated notable accuracy in speed detection, as evidenced by an RMSE value of 0.02 m/s, offering a technologically advanced, consistent, and non-intrusive method for tracking and estimating the locomotion speed of chickens. This research not only substantiates TAM as a potent tool for detailed poultry behavior analysis and monitoring but also illuminates its potential applicability in broader livestock monitoring scenarios, thereby contributing to the enhancement of animal welfare and management in poultry farming through automated, non-intrusive monitoring and analysis.

## 1. Introduction

Precision livestock farming (PLF) has rapidly evolved into a key field, merging modern technology with traditional animal farming to improve animal welfare and streamline production processes [1]. In poultry farming, it is essential to monitor and understand bird movement and behavior closely. This not only ensures the well-being of the animals but also helps improve production efficiency in a sustainable environment [2,3,4]. Chickens display a variety of behaviors, including different movement patterns, social interactions, and reactions to their surroundings. This requires advanced systems to track and analyze them effectively.

Deep learning, an advanced form of machine learning technology, is becoming a key tool for analyzing and predicting patterns in large and complex datasets [5,6]. In animal behavior studies, deep learning helps provide a detailed understanding of movement, interactions between species, and overall health [7]. In poultry farming, the use of deep learning offers more than just a glimpse into bird behaviors. It acts as a powerful tool to closely observe and track their activities [8]. Regarding post-observational monitoring, a slew of algorithms has found their footing in this domain, with you only look once (YOLO) being at the forefront. For instance, in large-scale poultry farms, the surveillance of thousands of chickens for health, activity, and behavioral patterns becomes pivotal [9]. YOLO’s rapid detection capabilities can identify early signs of disease or distress in chickens by recognizing subtle behavioral changes, thereby aiding farmers in timely interventions [5]. In addition, the proposed ChickTrack model uses deep learning to detect chickens, count them, and measure their movement paths, providing spatiotemporal data and identifying behavioral anomalies from videos and images [10]. However, while YOLO has shown commendable performance in a variety of scenarios, it is not exempt from limitations. For effective use in poultry farming, it demands rigorous training on domain-specific data to fine-tune its detection and tracking capabilities. The nuances of poultry behavior, their interactions, and variations in physical appearances require YOLO to be trained with vast and diverse datasets. But even with comprehensive training, the model might still face challenges in tracking individual entities within dense flocks, especially under varying environmental conditions [11]. It is in this context that the track anything model (TAM) emerges as a promising candidate. This research aims to harness the potential of TAM, enhancing its capabilities to not just track individual chickens in a flock, but to analyze their complex locomotion patterns in real-time [12,13,14]. By bridging the gaps left by previous models and incorporating the strengths of YOLO’s detection capabilities, TAM is poised to offer a holistic solution to the multifaceted challenges in poultry behavior analysis.

In this research, an innovative approach involving the strategic dyeing of chickens was adopted to augment the model’s capability to distinctly identify and track individual entities within the flock. The dyed chickens, exhibiting distinct and consistent coloration, serve as a unique identifier, facilitating improved tracking and identity preservation by the algorithms. The research further explores the adaptation of TAM, integrating a speed detection function, thereby providing a comprehensive tool for detailed poultry behavior analysis and monitoring. Through rigorous evaluations and comparative analyses, this research aims to underscore the efficacy and potential of TAM and its adaptation, TAM-*speed*, in providing a multifaceted solution for real-time poultry behavior tracking and analysis, thereby contributing to the advancement of precision livestock farming.

The objectives of this study were to: (1) develop a tracker model for monitoring the locomotion speed of individual chicks based on the TAM; (2) compare the TAM-*speed* model with state-of-the-art models such as YOLO, which are trained using images of chickens; and (3) test the performance of these newly developed models under various production conditions.

## 2. Materials and Methods

### 2.1. Data Acquisition

**Figure 1:**
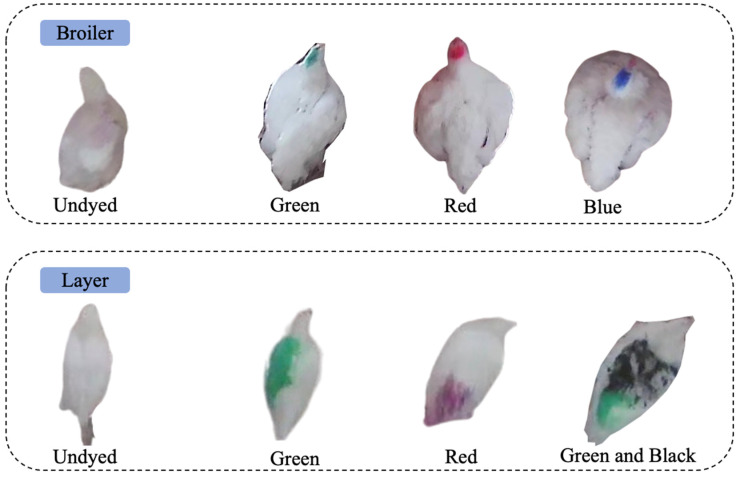
Contrast of dyed and undyed broilers and layers in experimental settings.

The dataset was obtained from two different experimental chicken houses (i.e., broilers and layers houses) in the Poultry Research Center at the University of Georgia (UGA), USA. Chickens were subjected to dyeing to assess the detection differences between dyed and undyed samples. Broilers were dyed with specific colors (green, red, and blue) and laying hens with another set (green, red, and black). Figure 1 illustrates the experimental chicken houses alongside their dyed counterparts. HD cameras (PRO-1080MSFB, Swann Communications, Santa Fe Springs, CA, USA) were affixed at a 3 m height on ceilings and walls in each room, capturing chicken behavior at 18 FPS with a 1440 × 1080 resolution. Lens maintenance involved weekly cleaning for clarity [15]. Image data were initially stored on Swann video recorders and subsequently transferred to HDDs (Western Digital Corporation, San Jose, CA, USA) at UGA’s Department of Poultry Science.

### 2.2. Marking Approach

Chickens were first subjected to a random selection process to determine which individuals would be used for the experiment. Once chosen, these chickens were dyed using the all-weather Quick Shot dye (LA-CO INDUSTRIES, INC, Elk Grove Village, IL, USA). The selection of dye colors aims to reduce feather flecking in dyed chickens [16]. The application process required a coordinated effort from a two-person team: while one individual gently held and restrained the bird to ensure its safety and ease of application, the other expertly applied the spray dye to the specific targeted areas on the chicken’s body, ensuring consistent and even coverage. This methodology was designed to minimize stress to the chickens while achieving a uniform application of the dye.

### 2.3. Model Innovation for Tracking Chickens

**Figure 2:**
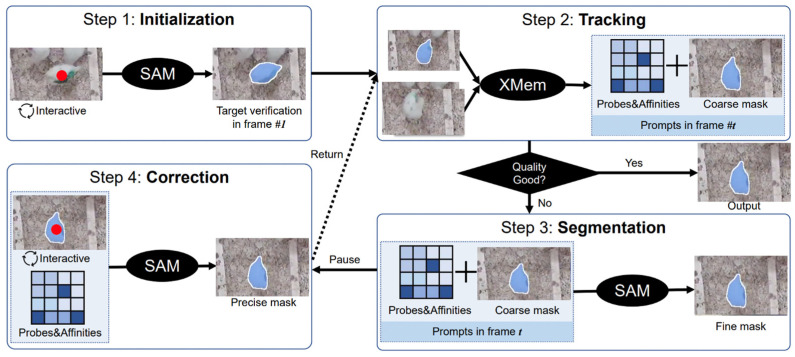
Pipeline of the track anything model (TAM) applied to chicken tracking [17].

In our study, we utilized the track anything model (TAM) to monitor chicken locomotion. Recognizing the versatility of TAM, we further enhanced it with a speed detection function, enabling the real-time measurement of each chicken’s velocity [17]. In the preprocessing phase, we utilized the XMem video object segmentation (VOS) technique to discern the masks of chickens across subsequent video frames [18]. XMem, renowned for its efficiency in standard scenarios, usually generated a predicted mask. However, when this forecast was suboptimal, our system captured both the prediction and key intermediate parameters, namely the probe and affinity. In instances where the mask quality fell below expectations, the SAM technique was harnessed to further refine the XMem-proposed mask using the said parameters as guidance. Recognizing the limitations of automated systems in intricate situations, we also factored in human oversight, allowing manual mask adjustments during real-time tracking to ensure optimal accuracy (Figure 2). The TAM architecture was structured such that preprocessed frames of size 1440 × 1080 served as input. Within the model, convolutional neurons were dedicated to extracting essential features like shape and color patterns. Crucially, by integrating TAM’s inherent capabilities with our innovations, we developed a layer that not only estimated chicken trajectories but also calculated their speed using the change in positional coordinates across frames and the associated time differential [19]. The output then presented both the chicken’s position and speed. We later benchmarked our enhanced TAM with a speed detection model (TAM-speed) against several state-of-the-art simple online and real-time tracking (SORT) models including observation-centric SORT (OC-SORT) [20], deep association metric SORT (DeepSORT) [21], ByteTrack [22], and StrongSORT [23], focusing on criteria such as tracking accuracy, speed measurement accuracy, frame processing rate, and model robustness in scenarios with dense poultry populations. For the models like OC-SORT, DeepSORT, ByteTrack, and StrongSORT, the comparison with TAM was primarily based on their tracking function. However, when it came to comparing TAM with you only look once version 5 (YOLOv5) and you only look once version 8 (YOLOv8), our motivation was distinct. YOLOv5 and YOLOv8 are renowned for their advanced segmentation capabilities, which are crucial for detailed object recognition and delineation in complex environments [24]. By comparing TAM with these YOLO versions, we aimed to evaluate how our model fares in terms of segmentation accuracy, efficiency, and reliability. Given the intricate patterns and overlapping scenarios often observed in poultry behavior, a robust segmentation function can significantly enhance the precision of tracking. Thus, understanding how TAM stands against the segmentation prowess of YOLOv5 and YOLOv8 can provide insights into potential areas of improvement and adaptation for our model. This adaptation of the TAM model aims to provide a comprehensive solution for real-time poultry behavior tracking, potentially paving the way for broader applications in livestock monitoring.

### 2.4. Methods of Speed Calculation in Chicken Tracking

**Figure 3:**
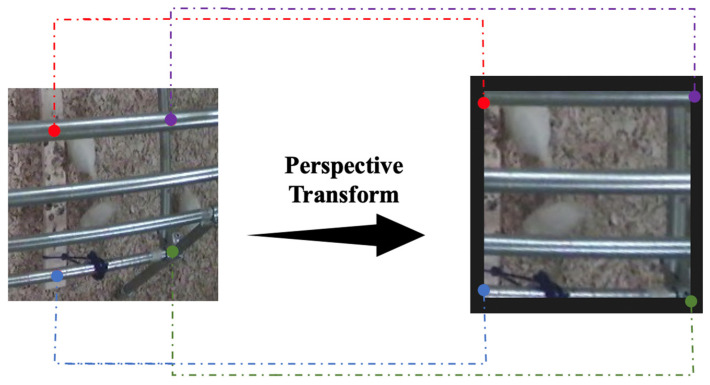
An illustration of a perspective transformation utilizing the OpenCV library.

Video analysis often encounters challenges in measuring the velocity of chickens due to distortions from camera perspectives. A video, which comprises continuous frames, enables the calculation of “pixel speed” by evaluating the chicken’s pixel displacement across frames within a time interval of 55.56 milliseconds (ms) at 18 FPS. However, the chicken’s motion can appear distorted in 2D frames due to 3D environmental dynamics. Our solution transforms the video frame to a top-down perspective, using open source computer vision library (OpenCV)‘s perspective transformation capabilities based on known rectangle coordinates in the original frame (Figure 3) [25]. This transformation eliminates horizontal discrepancies and relates vertical pixel shifts to the chicken’s actual distance traveled. Using this method and the time between frames, we were able to estimate individual chickens’ average velocity, which also indicates their real-time walking/running speed in closely spaced frames.

So, the equation to compute the actual speed V for chickens:

$$V=\frac{\Delta Y\times W}{\left(M-N\right)\times 55.56\;ms}$$

where:

**Δ*Y*** is the vertical pixel displacement of the chicken in the top-down view.
W is the actual physical distance represented by one pixel in the top-down view.
M and N are the frame numbers where the chicken’s position was recorded.

### 2.5. Model Evaluation Metrics

In our endeavor to optimize the track anything models for monitoring chickens’ locomotion, rigorous model evaluations were centered on specific metrics to ensure precise and consistent tracking of individual chickens across video sequences. The multiple objects tracking accuracy (MOTA) gauges the accuracy of the tracking model, considering discrepancies like false positives, misses, and identity switches. The identification F1 score (IDF1) becomes paramount in assessing the model’s proficiency in recognizing and consistently maintaining the identity of each chicken throughout sequences. IDF1 is computed as the harmonic mean of identification precision (IDP) and identification recall (IDR). IDP evaluates how many detections of a particular chicken identity are correct, while IDR calculates the proportion of actual detections for a chicken identity. Furthermore, the identity switches (IDS) metric quantifies instances when the system erroneously alters a chicken’s identity. The frames per second (FPS) metric serves as a testament to the model’s real-time monitoring efficacy, elucidating its processing speed [26]. When comparing TAM with YOLO, the mean Intersection over Union (mIoU) becomes essential. mIoU is a metric that evaluates the overlap between the predicted segmentation and the ground truth, providing insights into the model’s segmentation accuracy. In the context of TAM-speed detection accuracy, the root means square error (RMSE) is employed to quantify the model’s prediction accuracy in determining the chickens’ speed [27]. RMSE represents the square root of the average squared differences between the observed actual speed and the speed predicted by the model. Through this lens, the TAM-speed model’s efficacy in accurately detecting and predicting the chickens’ speed was rigorously evaluated, ensuring that the model not only proficiently tracks the chickens but also precisely gauges their speed, thereby providing a comprehensive tool for detailed poultry behavior analysis and monitoring. For each metric, we calculated the average from test results based on a test dataset across different models. These average values were then utilized to compare the performance among the various models.

$$MOTA=1-\frac{\left(FalsePositives+Misses+IdentitySwitches\right)}{TotalGroundTruthObjects}$$

$$IDF1=\frac{2\times (IDP\times IDR)}{\left(IDP\times IDR\right)}$$

$$IDP=\frac{TruePositives}{TruePositives+FalsePositives}$$

$$IDR=\frac{TruePositives}{TruePositives+Misses}$$

$$FPS=\frac{TotalFrames}{TotalTime(inseconds)}$$

$$mIoU=\frac{1}{N}\sum_{i=1}^{N}\left|\frac{{Prediction}_{i}\cap {GroundTruth}_{i}}{{Prediction}_{i}\cup {GroundTruth}_{i}}\right|$$

where N is the number of classes, ${Prediction}_{i}$ is the predicted segmentation for class i, and ${GroundTruth}_{i}$ is the ground truth for class i.

$$RMSE=\sqrt{\frac{1}{n}\sum_{i=1}^{n}{\left(y_{i}-{\hat{y}}_{i}\right)}^{2}}$$

where n is the total number of observations, $y_{i}$ is the actual speed of the chicken in the ith observation, and ${\hat{y}}_{i}$ is the predicted speed of the chicken in the ith observation.

## 3. Results

### 3.1. Comparison of Segmentation Approaches

**Figure 4:**
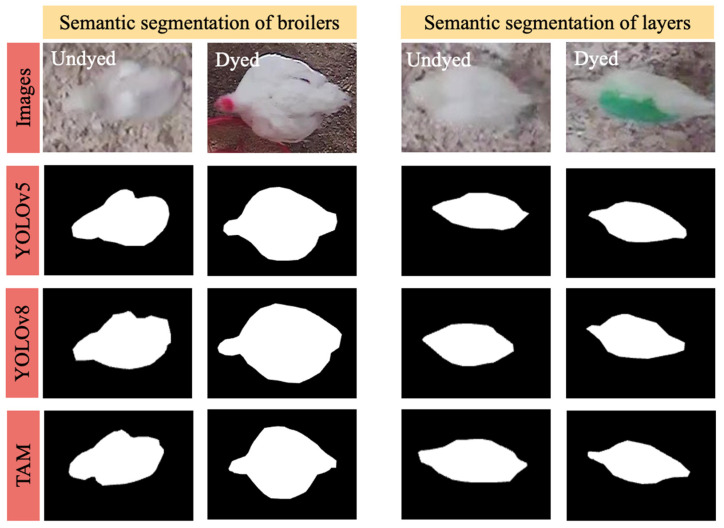
Visual comparison of segmentation results. YOLOv5 and YOLOv8 are compared with the TAM approach applied to diverse chicken datasets.

In our rigorous comparative analysis of segmentation methodologies for chicken tracking analysis, we evaluated YOLOv5, YOLOv8, and TAM. The chicken dataset, encompassing 1000 images, served as the foundation for this analysis. For the models necessitating training phases, specifically YOLOv5 and YOLOv8, a distribution of 600 images was allocated for training, 200 for validation, and the residual 200 for testing. The training regimen was orchestrated within a Python 3.7 environment, harnessing the capabilities of the PyTorch deep learning library, facilitated by an NVIDIA-SMI graphics card with a 16 GB capacity. Our segmentation efficacy evaluation spanned four distinct chicken categories: undyed broilers, undyed layers, dyed broilers, and dyed layers. A recurrent theme was the enhanced segmentation precision observed in dyed chickens, attributed to the pronounced color contrast introduced by dyeing, which counteracted the challenges posed by the chromatic resemblance between the chickens’ white plumage and the light brown litter. Despite the distinction between broilers and layers, no significant segmentation performance variance was observed, suggesting challenges predominantly driven by color rather than morphology. Among the methodologies, TAM, leveraging its pre-trained model, consistently outperformed both YOLOv5 and YOLOv8. This superiority can be attributed to TAM’s architectural robustness, its adeptness at high-dimensional feature extraction, and the efficacy of its pre-trained model [17], which potentially aligns better with the challenges presented by the chicken dataset. The forthcoming mIoU values in Table 1 will further detail TAM’s segmentation prowess, and a visual representation in Figure 4 underscores its potential as a leading choice for future chicken segmentation research.

### 3.2. Assessing the Performance of Chicken Tracking

Navigating through the intricate domain of chicken tracking, a comparative analysis was conducted, scrutinizing various algorithms, each harboring a unique blend of detection and tracking capabilities. The algorithms under the lens included YOLOv5+DeepSORT, YOLOv5+ByteTrack, YOLOv8+OC-SORT, YOLOv8+StrongSORT, and TAM, each meticulously paired to harness the strengths of YOLO’s object detection and the respective tracking proficiencies of the algorithms. YOLOv5 was paired with both DeepSORT and ByteTrack, leveraging its enhanced detection capabilities with DeepSORT’s deep association metrics and ByteTrack’s byte-level tracking, respectively, to maintain persistent identities of chickens, especially amidst occlusions and flock interactions. The dyed chickens, with their distinct colors, provided a vibrant scenario to evaluate the color-based tracking of these algorithms. The color distinction in dyed chickens inherently offers a unique identifier that facilitates improved tracking and identity preservation by the algorithms. In experiments, dyed chickens consistently demonstrated superior MOTA and IDF1 scores across all algorithms, indicating enhanced tracking accuracy and identity preservation, respectively. For instance, YOLOv5+DeepSORT exhibited a MOTA of 92.13% and IDF1 of 90.25% for dyed chickens, compared to slightly lower percentages for undyed ones. This trend was consistent across all algorithms, underscoring the pivotal role of distinct coloration in enhancing tracking performance [28].

In the case of YOLOv8, it was paired with OC-SORT and StrongSORT, evaluating their potential to minimize identity switches and maintain tracking accuracy amidst the dynamic and interactive poultry house environment. The algorithms were evaluated based on the TAM, ensuring a balanced assessment of both accuracy and computational efficiency, focusing on metrics such as MOTA, IDF1, and IDS. In the context of dyed chickens, YOLOv5+DeepSORT exhibited commendable tracking accuracy, leveraging the color features effectively, yet faced challenges in maintaining identities during occlusions. YOLOv5+ByteTrack showcased robustness in handling identity switches but at a computational cost, reflected in a lower FPS. YOLOv8+OC-SORT demonstrated enhanced tracking accuracy in scenarios of chicken interactions and occlusions due to its observation-centric approach, while YOLOv8+StrongSORT, maintaining a high MOTA, faced challenges in dense chicken populations, leading to a higher IDS [29]. Considering the comparative values provided in experiments and illustrated in Table 2, TAM emerges as the superior model, substantiating its position as the best model among those evaluated. It boasts the highest MOTA, indicating the highest accuracy in tracking while minimizing misses and false positives. It achieves the highest IDF1 score, showcasing its proficiency in maintaining consistent identities throughout the tracking period. Furthermore, TAM registers the lowest number of Identity Switches (IDS), reflecting its capability to preserve identities accurately across frames with minimal switches. This unique capability of TAM to provide accurate tracking alongside its superior tracking accuracy underscores its unparalleled utility in comprehensive poultry behavior analysis, thereby substantiating its position as the best model among the ones evaluated. This assessment reveals a trade-off between tracking accuracy and computational efficiency, suggesting that advancements in TAM could potentially enhance poultry tracking in future applications.

### 3.3. Evaluating Velocity Measurement

**Figure 5:**
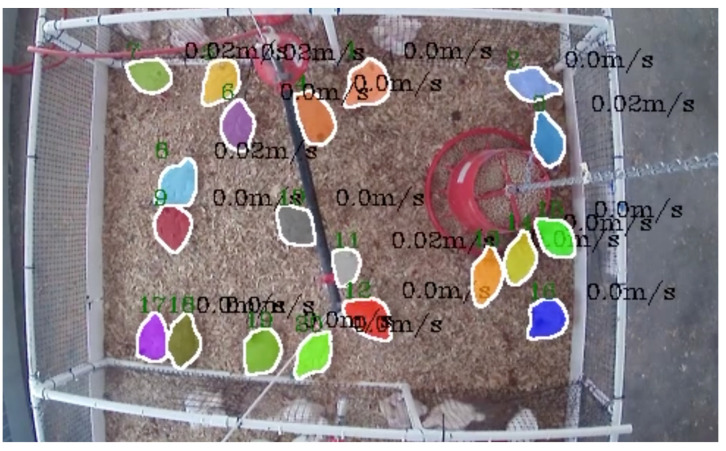
Track and detection speed of broilers (the green number indicates the tracking number, while the black number represents speed).

In the meticulous pursuit of accurate and reliable chicken tracking, TAM-*speed* has been subjected to a thorough evaluation, particularly focusing on its capability to accurately detect and quantify the speed of chickens within a controlled environment. In our experiments, where the average speed of the chickens was measured to be 0.05 m/s, the precision with which TAM-*speed* could predict and validate these speed measurements became paramount. Utilizing the RMSE as a pivotal metric to quantify the average discrepancies between the speeds predicted by TAM-*speed* and the actual observed speeds, a comprehensive analysis was conducted. Given that RMSE provides a high penalty for larger errors, it serves as a stringent metric, ensuring that the model’s predictions are not only accurate on average but also do not deviate significantly in individual predictions. In our analysis, dyed chickens, with their distinct and consistent coloration, provided a somewhat stable basis for the tracking algorithm to latch onto, potentially minimizing the instances where tracking was lost or inaccurately assigned. The RMSE for dyed chickens was recorded at a laudable 0.02 m/s, indicating a high degree of accuracy in speed detection. The distinct coloration likely assisted the model in maintaining a consistent track, thereby enabling more accurate speed calculations over a sequence of frames. Conversely, undyed chickens, with their more variable and less distinct visual features, posed a slightly more complex scenario for TAM-*speed*. The RMSE for undyed chickens was marginally higher, recorded at 0.025 m/s. This subtle elevation in error might be attributed to the challenges in maintaining consistent tracking amidst the visually similar undyed chickens, potentially leading to brief losses in tracking or misidentifications, which in turn, could slightly skew the speed calculations [30]. Despite these discrepancies, it is crucial to note that in the dynamic and somewhat unpredictable environment of a poultry house, numerous variables can influence the chickens’ speed, such as their age, size, and overall health, as well as external factors like lighting and noise levels. Despite the challenges, TAM-*speed* has showcased a commendable capability in speed detection, providing predictions that, while subject to error, still provide valuable insight into the locomotion and behavior of the chickens. The utility of such a model extends beyond mere speed detection, offering potential insights into the health and well-being of the poultry by monitoring their mobility and activity levels [31]. In conclusion, while TAM-*speed* demonstrates a notable accuracy in speed detection, it is imperative to continually refine the model, considering the myriad of variables that can influence the speed and behavior of chickens. Future iterations of the model might benefit from additional training data, encompassing a wider range of scenarios and conditions, to further enhance its predictive accuracy and reliability in diverse poultry house environments. Table 3 summarizes the velocity changes among dyed and undyed chickens. Figure 5 displays a visualization of speed and track detected by TAM-*speed*.

## 4. Discussion

### 4.1. Chicken Segmentation Approaches

In the present exploration, TAM has notably eclipsed both YOLOv5 and YOLOv8 in a variety of tracking tasks, particularly those involving chickens in dyed condition. Specifically, TAM’s integrated mode, which amalgamates tracking and speed measurement, has showcased unparalleled precision across diverse tracking scenarios. This exemplary performance can be attributed to several pivotal factors. Firstly, TAM utilizes a specialized tracking mechanism that adeptly captures intricate movement patterns and complex trajectories, enabling it to focus on pertinent features and trajectories, thereby facilitating more accurate tracking. Moreover, it is worth noting that TAM surpassed other models without necessitating additional training or extensive fine-tuning. This implies that the architecture and design of TAM inherently possess robust tracking capabilities, negating the need for exhaustive model adjustments or specialized training datasets. This inherent proficiency not only underscores TAM as a more practical and effective option for tracking applications but also highlights its potential to be applied in various poultry tracking scenarios without the need for exhaustive model adjustments or specialized training datasets. In addition, the segmentation of dyed chickens consistently exhibited superior performance across all algorithms when compared to undyed chickens. This can be attributed to the distinct colors of the dyed chickens, which provide a more discernible feature for the model to track, thereby reducing identity switches and enhancing tracking accuracy [32]. This nuanced capability of TAM to adeptly manage variations in object features further solidifies its position as a versatile and reliable model for chicken tracking applications. Comparing the tracking of whole chickens, it was observed that tracking dyed chickens demonstrated superior performance across all metrics. This is because tracking dyed chickens may provide additional distinctive features for the model to latch onto, thereby facilitating improved tracking results. The tracking of a dyed chicken provides a more comprehensive understanding of the object by capturing its overall shape and structure, which facilitates improved tracking results. Table 4 presents a comparative analysis of TAM with various research studies in the domain of chicken tracking using computer vision. For instance, EfficientNet-B0 achieved a mIoU of 89.34% in a study involving the segmentation of meat carcasses using a dataset of 108,296 images [6]. Similarly, MSAnet secured a mIoU of 87.7% for segmenting caged poultry across a 300-image dataset [2], while Mask R-CNN recorded a mIoU between 83.6% and 88.8% for segmenting hens in a 1700-image dataset [33]. Contrarily, TAM demonstrated a mIoU of 93.12% for poultry tracking, potentially outperforming other methods even without a specialized target dataset. This highlights TAM’s ability to accurately trace chicken movements within images and underscores its efficacy and potential applicability in broader computer vision tasks related to chicken tracking.

### 4.2. The Precision of Velocity Measurement in Poultry Tracking

In the realm of poultry tracking, the implementation of speed detection, particularly through computer vision, remains a relatively unexplored territory. The TAM-*speed* model, however, has emerged as a pioneering approach in this domain, offering a novel perspective in estimating the velocity of broiler and layers. This model, while primarily focused on tracking, also encapsulates the capability to measure speed, providing a dual functionality that is both innovative and crucial for comprehensive poultry behavior analysis. In contrast, the field of vehicle speed detection has witnessed substantial advancements, with numerous methodologies being developed and refined over the years. A common approach within this domain involves the utilization of a perspective transformer, which aids in estimating the speed of vehicles by analyzing the change in position of a vehicle over consecutive frames, considering the camera’s perspective [26,34]. This method, while effective for vehicles, presents unique challenges when applied to poultry due to the erratic and non-linear movement patterns exhibited by chickens. Comparatively, other methods of speed detection in poultry have traditionally relied on wearable equipment or radio speed detection techniques. Wearable devices, while providing accurate data, may influence the natural behavior and movement of the chickens due to the physical burden and potential stress induced by the equipment [35,36]. On the other hand, radio speed detection, which typically involves tracking the radio frequency identification (RFID) tags attached to the chickens, may offer valuable data but is often constrained by its dependency on the proximity and orientation of the RFID tags, potentially limiting the accuracy and consistency of the data collected [37,38,39]. TAM-*speed*, in this context, offers a non-intrusive, consistent, and technologically advanced method of not only tracking but also estimating the speed of chickens without the need for physical contact or proximity-based technology. It leverages computer vision to analyze movement and estimate speed, providing a wealth of data that are both accurate and comprehensive, without influencing the natural behaviors of the poultry.

### 4.3. Limitations and Future Works

TAM and its derivative, TAM-*speed*, exhibit a notable limitation in their substantial computational and memory demands, especially when applied to scenarios involving the tracking of numerous entities over extended durations. In specific test cases, even when utilizing the robust NVIDIA A100 GPU, which is equipped with a substantial 96 GB of memory and is renowned for its computational prowess, the models encountered difficulties in sustaining tracking for periods exceeding 2 min, particularly when tasked with simultaneously tracking more than 20 individual chickens. This computational demand not only restricts the duration and scale of tracking but also poses significant barriers to its application in real-world, large-scale poultry farms where continuous monitoring of larger flocks is imperative for effective management and research.

In future endeavors, leveraging distributed computing can mitigate TAM’s computational demands, enabling the analysis of larger poultry populations and extended tracking durations. Additionally, incorporating edge computing strategies, where initial data processing occurs on local devices, could alleviate the computational load on the central model, ensuring efficient and timely poultry behavior analysis. Furthermore, implementing adaptive sampling techniques, which dynamically adjust the TAM model’s sampling rate based on scene complexity, could optimize computational resource allocation, ensuring detailed analyses during complex behaviors while conserving resources during simpler scenarios [40].

## 5. Conclusions

The track anything model and its adaptation, TAM-*speed*, have emerged as potent tools for analyzing chicken locomotion and behavior, demonstrating superior performance in tracking and segmenting dyed chickens compared to other models like YOLOv5 and YOLOv8. TAM achieved a mean Intersection over Union (mIoU) of up to 95.13%, showcasing its architectural robustness and effective pre-trained model. Furthermore, TAM-*speed* exhibited commendable speed detection capabilities, with an RMSE of 0.02 m/s for dyed chickens, providing valuable insights into poultry behavior and potential health indicators. This research underscores TAM’s potential as a multifaceted tool for comprehensive poultry behavior analysis without requiring extensive training or fine-tuning, paving the way for advanced applications in precision livestock farming.

## Figures and Tables

**Table 1:** A comparison of TAM and YOLOv5 and YOLOv8 in terms of mean intersection over union (mIoU).

Method	Semantic Segmentation of Broilers		Semantic Segmentation of Layers	
	Undyed	Dyed	Undyed	Dyed
YOLOv5	81.26	85.63	80.79	85.51
YOLOv8	83.44	86.91	82.59	87.72
TAM	93.15	95.13	92.17	94.82

Notes: Track anything model (TAM) and you only look once (YOLO).

**Table 2:** Comparative analysis of tracking algorithms for dyed and undyed chickens.

Algorithm	Condition	MOTA (%)	IDF1 (%)	IDS	FPS
YOLOv5+DeepSORT	Dyed	92.13	90.25	15	18
YOLOv5+DeepSORT	Undyed	88.47	86.32	25	18
YOLOv5+ByteTrack	Dyed	93.21	91.47	14	15
YOLOv5+ByteTrack	Undyed	89.36	87.14	22	15
YOLOv8+OC-SORT	Dyed	95.67	93.12	12	17
YOLOv8+OC-SORT	Undyed	91.78	89.12	20	17
YOLOv8+StrongSORT	Dyed	94.56	92.34	13	18
YOLOv8+StrongSORT	Undyed	90.12	88.45	23	18
TAM-*speed*	Dyed	97.45	95.67	10	16
TAM-*speed*	Undyed	94.78	92.34	18	16

Notes: Track anything model (TAM), you only look once (YOLO), multiple objects tracking accuracy (MOTA), identification F1 score (IDF1), identity switches (IDS), and frames per second (FPS).

**Table 3:** Comparative analysis of velocity for dyed and undyed chickens.

Algorithm	Condition	RMSE (m/s)	Velocity Range (m/s)
TAM-*speed*	Dyed	0.02	0.00–0.21
TAM-*speed*	Undyed	0.025	0.00–0.21

**Table 4:** Comparison of different methods on segmentation accuracy.

Methods	Dataset (Constructed by Authors)		mIoU (%)
	Number	Type	
EfficientNet-B0	108,296	meat carcasses	89.34
MSAnet	300	caged chickens	87.7
mask R-CNN	1700	hens	83.6–88.7
TAM (this study)	/	/	93.12

## Data Availability

The data presented in this study are available on reasonable request from the corresponding author.
